# Assessing the correlation between STAR health risk forecasts with actual health emergencies in West and Central Africa: a three-year analysis

**DOI:** 10.3389/fpubh.2025.1728832

**Published:** 2026-01-07

**Authors:** Daniel Yota, Christian Eric Massidi, Ambrose Talisuna, Omer Njajou Tchikamgoua

**Affiliations:** 1 World Health Organization, Dakar, Senegal; 2 World Health Organization, Nairobi, Kenya; 3 Public Health Department, Université des Montagnes, Bangangté, Cameroon

**Keywords:** emergency preparedness, occurrence, prediction, risk assessment, STAR tool

## Abstract

**Introduction:**

This study evaluates the Strategic Tool for Assessing Risks (STAR), developed by the World Health Organization (WHO), for its effectiveness in predicting public health emergencies across West and Central Africa during the period 2022–2024. STAR applies a composite risk scoring system based on four dimensions likelihood, severity, vulnerability, and coping capacity to classify hazards such as measles, cholera, and meningitis into five risk categories ranging from very low to very high.

**Methods:**

Using a retrospective observational design, the study integrates quantitative outbreak data with qualitative assessments of preparedness actions across nine countries. The analysis demonstrates that STAR’s predictive accuracy varies significantly by hazard and context.

**Results:**

Meningitis forecasts were consistently accurate, primarily due to the disease’s strong seasonality and well-established epidemiological patterns in the African meningitis belt. In contrast, predictions for measles and cholera were less reliable, influenced by fluctuating immunization coverage, socio-political instability, and environmental factors such as water and sanitation conditions. Case studies illustrate these discrepancies: Burkina Faso’s cholera risk was overestimated, resulting in zero reported cases despite a high-risk classification, while Guinea’s measles outbreak closely matched STAR’s high-risk prediction. The findings also highlight that effective preparedness measures, including vaccination campaigns, hygiene promotion, and cross-border coordination, can mitigate high-risk scenarios, as observed in Gabon and Burkina Faso. Key themes emerging from the analysis include STAR’s strength in forecasting predictable hazards and its limitations due to static inputs and low geographic granularity.

**Discussion:**

While STAR is not a statistical forecasting model, its participatory, multi-sectoral approach provides strategic value by guiding planning, prioritization, and resource allocation for health emergency preparedness. It enables countries to optimize limited resources, prioritize highrisk hazards (scores 16–25), and implement preventive actions. Recommendations for improvement include recalibrating scoring parameters, integrating real-time surveillance and climate data, enhancing seasonality modeling, and increasing geographic resolution. When combined with dynamic data systems and collaborative efforts, STAR remains a critical strategic tool for strengthening regional public health resilience and supporting WHO’s Health Emergency Framework and all-hazards preparedness planning.

## 1 Introduction

Most countries will likely face a major emergency about every five years, and many will experience seasonal hazards like cyclones, flooding, and disease outbreaks. While countries may aim to address all risks quickly and effectively, risk-based strategies can help optimize resources and prioritize actions to prepare for potential emergencies. Suppose outbreaks and disasters are not managed effectively. In that case, they can cause significant short- and long-term impacts at the individual, community, national, and global levels (1) resulting in poor psychological well-being, forced displacement of the affected population, damage health infrastructure and ecosystem degradation.

Adopting a risk-based approach for managing health emergencies and reducing risks, countries must first identify hazards and evaluate their risk levels within the country. The risk assessment results enable effective planning and prioritization of efforts to prevent better, mitigate, detect early, prepare for, be operationally ready for, respond to, and recover from a health emergency or disaster (2).

The Strategic Tool for Assessing Risks (STAR) offers an easy-to-use, comprehensive toolkit and approach to enabling countries and regions to conduct a strategic, rapid, and evidence-based assessment of public health risks for planning and prioritizing health emergency preparedness and disaster risk management activities (3). In this regard, several countries of West Central Africa have assessed their risk profiling and mapping using the STAR tool WHO developed by WHO. From 2021 to 2023, 16 countries organized STAR workshops at the national or sub-national levels to prepare to respond to any potential health emergency. With the results of the strategic risk assessment, those countries were able to apply evidence to inform country planning, prioritize key actions for rapid scale-up of capabilities for high risks, and rationalize and effectively use limited resources to strengthen health emergency and disaster risk management capacities in the context of competing priorities. Thus, it is recommended that countries conduct this exercise every 2 to 3 years to update their health risk profiling and get better prepared to face the next emergency.

**Figure 1:**
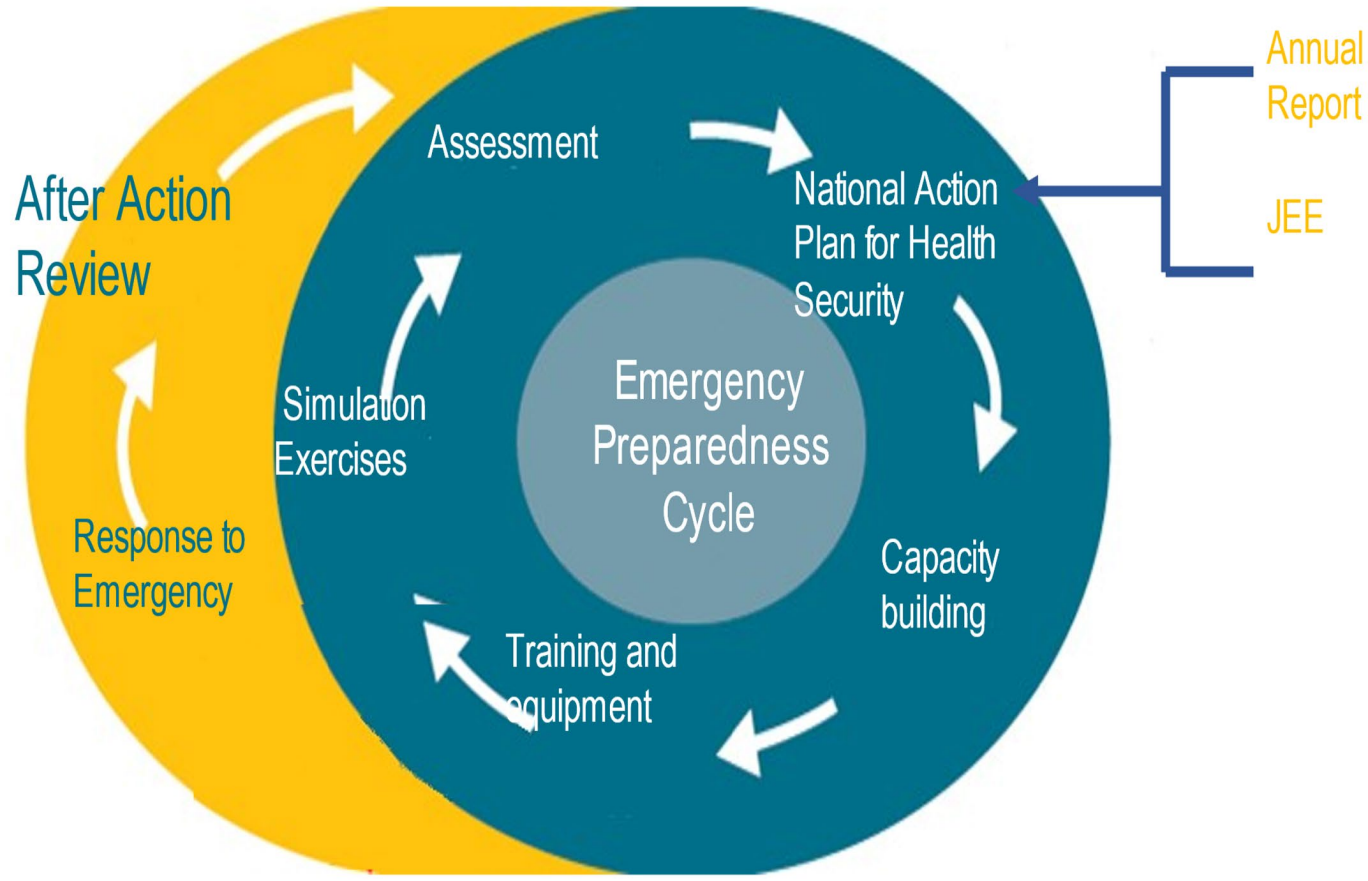
Emergency preparedness cycle.

The objective of this study is to analyse the correlation between the prediction of hazards from the STAR assessments and the actual occurrence of health emergencies, such as disease outbreaks and other crises, over the period of 3 years, after which health risk profiling expires and requires update. This will enable us to assess the strengths and limitations of the STAR tool in forecasting health risks and propose recommendations for its improvement and integration into national health security systems (see Figure 1).

## 2 Materials and methods

### 2.1 Purpose

The purpose of this research is to evaluate the predictive accuracy of the STAR (Strategic Tool for Analysis of Risks) framework by comparing its health hazard risk classifications with actual emergency occurrences across selected countries in West and Central Africa over a three-year period.

The STAR tool does not generate numerical predictions of case counts. Its output is a composite risk score derived from four dimensions: Likelihood, Severity, Vulnerability, and Coping Capacity, all summed to produce a total score ranging from 4 to 25. This score is then classified into five categories: Very Low, Low, Moderate, High, and Very High. For visualization purposes, these categorical risk levels were converted into numeric codes (e.g., Risk 1 = Very Low, Risk 2 = Moderate, Risk 3 = High). These numeric codes were plotted alongside actual case counts in figures and tables to illustrate correlation trends. They do not represent predicted case numbers but rather the relative risk classification assigned by STAR.

### 2.2 Objectives

1. To compare STAR risk classifications with actual outbreak data for key hazards (Measles, Cholera, Meningitis).
2. To evaluate the effectiveness of preparedness measures in mitigating predicted risks.
3. Identify patterns and gaps in STAR’s predictive accuracy across different contexts.
4. To provide recommendations for enhancing risk assessment and emergency preparedness strategies.

### 2.3 Study type

As a retrospective observational study, it analyzes existing data from past events to identify patterns, correlations, or outcomes without manipulating variables or intervening in real time. In this context, the study examines historical health risk predictions made using the STAR Tool. It compares them with actual emergency occurrences across selected countries in West and Central Africa from 2022 to 2024. A mixed-methods approach combines quantitative analysis of outbreak data and a review of qualitative preparedness measures.

### 2.4 Study period

The study is conducted on health risk profiling carried out from January 2022 to December 2024 using STAR.

### 2.5 Geographic scope

The study focuses on nine countries in West and Central Africa to evaluate the effectiveness of the STAR (Strategic Tool for Assessing Risks) in various contexts, specifically in West Africa (including Guinea, Senegal, Mali, Burkina Faso, and Nigeria) and Central Africa (comprising Gabon, Cameroon, the Republic of Congo, and the Central African Republic).

The selection ensures broad geographical and contextual representativeness across the two sub-regions: by including countries from both West Africa and Central Africa, the study considers the distinct epidemiological settings and regional differences in health systems and WHO Regional Office structures (AFRO). Taking into account the diverse environmental and security contexts, the sample encompasses a wide range of geographical and political environments, including coastal states (e.g., Gabon, Senegal, Nigeria), landlocked countries (e.g., Mali, Burkina Faso, Central African Republic), Sahelian zones (Mali, Burkina Faso), which face high levels of insecurity and humanitarian crises that directly affect health vulnerability, and forest and equatorial zones (e.g., Cameroon, Congo, Gabon), which have different disease profiles and environmental risks.

Regarding varying disease profiles, this diverse geographical selection ensures that the STAR tool’s predictive capabilities are tested across a full range of regionally common hazards, such as meningitis (seasonal in the Sahelian belt), cholera (linked to environmental and sanitation factors, often near coastlines or major rivers), and measles (related to immunization coverage in all settings). Additionally, including countries from both Francophone and Anglophone regions, such as Nigeria, helps evaluate the tool’s performance within different health administration models and levels of preparedness.

### 2.6 Data sources and normalization

The outbreak data used in the study have been verified by combining standardized and independent official sources. The methodology explicitly addressed verification by implementing safeguards to ensure objectivity and scientific integrity of the findings, considering potential conflicts of interest. The outbreak data was sourced and verified using official and standardized documents and systems such as National Surveillance Data (obtained directly from the Ministries of Health), International Reports (outbreak reports and situation updates from WHO, Ministries of Health, and humanitarian platforms), Independent Data sources (national surveillance systems and WHO situation reports to confirm outbreak occurrences as a way to reduce bias), and official documentation from institutional repositories, government surveillance systems, and international health agencies with appropriate permissions. This triangulation process, which merges quantitative outbreak data from surveillance systems with qualitative assessments from official reports, served as a key measure to validate the actual occurrence of health emergencies.

### 2.7 Key variables

The study uses a retrospective observational design. The analysis compares two main variables namely the predicted Risk Level and the actual occurrence. The comparison relies on interpreting whether a high-risk prediction visually corresponds to a high number of raw cases, rather than adjusting the case data for population size, reporting capacity, or other factors that normalization would account for. Triangulation (merging quantitative outbreak data from surveillance systems with qualitative assessments from official reports) was a key measure to validate the actual occurrence, which is a measure of verification rather than statistical normalization.

### 2.8 Analytical method utilized to correlate STAR risk ratings with epidemic data

The study used a retrospective, observational, mixed-methods design to assess the correlation between STAR-predicted risk levels and actual outbreak occurrences across nine countries in West and Central Africa (2022–2024).

#### 2.8.1 Independent and dependent variables are used in the research

Independent Variables are STAR-predicted risk level for each hazard (Measles, Cholera, Meningitis), categorized as Very Low (1–5), Low (6–10), Moderate (11–15), High (16–20), and Very High (21–25) whereas dependent variables correspond to actual epidemiological data, including outbreaks, case counts, deaths, and geographic spread.

### 2.9 Expected outcomes

Identification of strengths and limitations in STAR’s predictive capacity, recommendations for improving risk assessment and preparedness planning, Evidence to support strategic resource allocation and early warning systems are the expected products of the research at the attention of WHO Regional Office for Africa, Ministries of Health, Humanitarian and development partners, and Emergency preparedness and response teams.

### 2.10 Risk scoring framework

Each hazard is assessed using a composite risk score, which is the sum of four key dimensions:

**Table:** 

Component	Description
Likelihood	Probability that the hazard will occur in each month.
Severity	Expected impact if the hazard occurs (e.g., mortality, morbidity).
Vulnerability	Susceptibility of the population (e.g., age, immunization status, poverty).
Coping capacity	Ability of the health system to respond (e.g., infrastructure, workforce).

The Strategic Tool for Assessing Risks (STAR) primarily categorizes the risk rather than giving actual numbers of predicted cases.

### 2.11 Risk scoring framework (revised)

The Strategic Tool for Assessing Risks (STAR) provides categorical risk assessments rather than numerical case predictions. Its primary output is a composite risk score derived from four dimensions Likelihood, Severity, Vulnerability, and Coping Capacity—summed to produce a total score ranging from 4 to 25. This score is classified into five categories:

- 1–5: very low risk
- 6–10: low risk
- 11–15: moderate risk
- 16–20: high risk
- 21–25: very high risk

For ease of visualization in tables and figures, STAR’s descriptive risk categories were converted into numeric codes such as Risk 1 for Very Low; Risk 2 for Moderate and Risk 3 for High. This coding was applied only for graphical representation and does not alter STAR’s official classification system (very low, low, moderate, high, very high).

## 3 Results

This analysis compared STAR’s predicted risk classifications with actual outbreak data for measles, cholera, and meningitis across nine countries in West and Central Africa from 2022 to 2024. The objective was to assess whether higher risk levels corresponded to higher observed case counts.

### 3.1 Overall findings

For meningitis, Predictions demonstrated the highest consistency with actual cases. Countries classified as Moderate to High risk generally experienced seasonal outbreaks, confirming STAR’s reliability for hazards with predictable drivers. As concerns measles: Correlation was variable. In some countries (e.g., Cameroon and Côte d’Ivoire), high-risk classifications aligned with major outbreaks. However, in others (e.g., Burkina Faso), STAR underestimated risk due to dynamic factors such as declining immunization coverage and service disruptions. Regarding cholera: Predictions were least reliable. Several countries classified as High risk reported zero cases, likely due to effective interventions or environmental conditions not captured by STAR. Conversely, Cameroon experienced a significant outbreak despite similar risk classification, indicating limitations in accounting for local variability.

#### 3.1.1 Cumulative raw data: predicted risk vs. actual cases (2022–2024)

The table aggregates the cumulative total actual cases reported over the three-year study period against the predicted risk level assigned by the STAR tool for key hazards in the countries with the most detailed comparative data (Cameroon and Côte d’Ivoire), with estimated totals for the remaining countries included in the final analysis (see Tables 1, 2).

**Table 1:** Cumulative comparison of STAR-predicted risk levels and observed outbreak case counts for selected hazards (2022–2024).

Country	Hazard	STAR predicted risk level	Actual cases (2022–2024)	Correlation/key finding
Cameroon	Measles	High (risk 3)	10,404	The large number of validated cases predicted a high risk.
	Cholera	High (risk 3)	1,992	The prediction slightly underestimated the severity of the 2022 outbreak.
	Meningitis	Moderate (risk 2)	85	Predictions generally aligned with low but persistent seasonal trends.
Côte d’Ivoire	Measles	High (risk 3)	2,154	Prediction was matched, particularly with the sharp rise in 2024.
	Cholera	High (risk 3)	0	No reported cases, despite a high predicted risk, suggesting either effective interventions or gaps in surveillance.
	Meningitis	Moderate (risk 2)	23	Low, consistent case count aligned with the moderate risk classification.

Interpretations indicate general agreement between predicted risk categories and actual case numbers for Cameroon and Côte d’Ivoire.

**Table 2:** Comparison of meningitis STAR-predicted risk levels and observed cases across countries, highlighting general alignment with seasonal patterns.

Country	STAR predicted risk level	Actual cases (2022–2024)	Correlation/key finding
Cameroon	Moderate (risk 2)	85	Predicted risk generally aligned with observed seasonal trends
Côte d’Ivoire	Moderate (risk 2)	23	Predicted risk generally aligned with observed seasonal trends
Burkina Faso	High	Not available	No case data; interpretation based on historical trends and preparedness actions
Central African Republic	High	Not available	No case data; interpretation based on historical trends and preparedness actions
Gabon	High	Not Available	No case data; interpretation based on STAR classification and preparedness measures
Guinea	High	Not available (moderate activity reported qualitatively)	Interpretation based on qualitative reports of moderate activity
Mali	High	Not available	No case data; interpretation based on historical trends and preparedness actions
Nigeria	Not applicable	Not available	Data gap: Insufficient data for meaningful correlation analysis

“Not available” = data missing or not reported; “not applicable” = no STAR risk level assigned for this hazard/country.

The results of the analysis, which compared the STAR Predicted Risk Level (a categorical score) with Actual Cases (raw counts), are summarized above for each of the three hazards across the nine studied countries:

The predictive accuracy varied significantly, with Meningitis showing the highest consistency and Cholera and Measles showing more discrepancies (see Tables 3, 4).

**Table 3:** Comparison of cholera STAR-predicted risk levels and observed cases.

Country	STAR predicted risk level	Actual cases (2022–2024)	Correlation/key finding
Cameroon	High (risk 3)	1,992	Predicted risk did not fully reflect observed outbreak size
Côte d’Ivoire	High (risk 3)	0	Predicted risk was higher than observed cases
Burkina Faso	Moderate	0	Predicted risk was higher than observed cases
Central African Republic	Moderate	0	Predicted risk was higher than observed cases; likely due to preparedness measures.
Republic of Congo	Very high	21 confirmed cases	Predicted risk was higher than observed cases; likely due to preparedness measures.
Gabon	Very low	0	Predicted risk generally aligned with observed seasonal trends
Guinea	Very low	0	Predicted risk generally aligned with observed seasonal trends
Mali	Low	0	Predicted risk generally aligned with observed seasonal trends
Nigeria	High	Reported cases (data incomplete)	Predicted risk generally aligned with observed seasonal trends
Burundi	Not applicable	0 reported cases	Discrepancy: Predicted risks did not match actual zero cases, possibly due to underreporting. Burundi did not identify cholera as a potential hazard during risk profiling

This table highlights areas of agreement and divergence influenced by interventions and environmental factors.

**Table 4:** Comparison of measles STAR-predicted risk levels and observed cases, illustrating variability linked to immunization coverage and service disruptions.

Country	STAR predicted risk level	Actual cases (2022–2024)	Correlation/key finding
Cameroon	High (risk 3)	10,404	Predicted risk generally aligned with observed seasonal trends
Côte d’Ivoire	High (risk 3)	2,154	Predicted risk generally aligned with observed seasonal trends
Burkina Faso	Moderate/high	5,688 (Surge in 2024)	Predicted risk did not fully reflect observed outbreak size
Central African Republic	High	Reported cases (data incomplete)	Predicted risk generally aligned with observed seasonal trends
Gabon	Very high	Not available	No case data; interpretation based on preparedness actions
Guinea	High	Major outbreak reported	Predicted risk generally aligned with observed seasonal trends
Mali	High risk	Reported cases with outbreak	Predicted risk generally aligned with observed seasonal trends
Nigeria	Moderate	Reported cases with outbreak	Variable Accuracy: Exhibited a moderate correlation but high variability, suggesting unpredictability in outbreaks.

A cross-country analysis of the data reveals distinct patterns in STAR’s predictive performance across the three hazards:

- Meningitis: high accuracy: predictions for meningitis demonstrated the greatest consistency with actual cases across all participating countries. The high predictive accuracy is primarily due to the disease’s strong seasonality, which peaks during the dry season, and well-established epidemiological patterns in the African meningitis belt. In countries such as Burkina Faso, actual cases closely followed forecasts.
- Cholera: mixed/poor accuracy: cholera predictions were the least reliable, often not reflecting actual conditions; Overprediction: In countries like Burkina Faso, the Central African Republic (CAR), and Côte d’Ivoire, a high or moderate predicted risk led to zero or near-zero actual cases, possibly due to effective preventative measures (WASH interventions) or overestimating risk factors; Underestimation: Conversely, the high predicted risk in Cameroon still underestimated the severity of the 2022 outbreak, which had over 1,700 cases.
- Measles: variable accuracy: measles demonstrated a moderate correlation but high variability, influenced heavily by changing immunization coverage and socio-political factors; Where predictions were accurate (e.g., Cameroon and Côte d’Ivoire), the high-risk classification was validated by major outbreaks (10,404 and 2,154 cases, respectively); Discrepancies arose due to underestimation of epidemics stemming from low immunization rates and COVID-19-related service disruptions, as seen by the surge in cases in Burkina Faso in 2024.

This quantitative synthesis confirms that while STAR is effective for predictable, seasonal hazards, its reliance on static inputs and inability to capture real-time, dynamic factors (such as falling vaccination rates or effective local interventions) limit its precision for rapidly evolving or intervention-sensitive diseases.

#### 3.1.2 Burkina Faso: disease risk forecasting and epidemiological reality

Over the past three years (2021–2023), Burkina Faso has faced a complex and evolving public health landscape shaped by infectious disease outbreaks, climate-related health risks, and efforts to strengthen epidemic preparedness. The analysis of disease risk predictions versus actual case data in Burkina Faso reveals a nuanced picture of public health dynamics and the effectiveness of forecasting models.

Despite the high risk classification, actual cases remained near zero due to effective preparedness measures, including early detection and cross-border coordination. Predictions were pretty accurate, with actual cases closely tracking forecasts, primarily due to outbreaks from serogroups W, X, and C. The annotation highlights the success of MenAfriVac® against serogroup A, but outbreaks from other serogroups (W, X, C) still occur. Surveillance has improved, but vaccine gaps remain a challenge. Low vaccination coverage led to a significant outbreak in 2024, confirmed by actual cases. Predicted case numbers fluctuate as vaccination trends change. Cases sharply decrease in 2022 and then spike again in 2024–2025 due to low immunization rates and COVID-19 disruptions.

##### 3.1.2.1 Example of Cameroon

See Figure 2.

**Figure 2:**
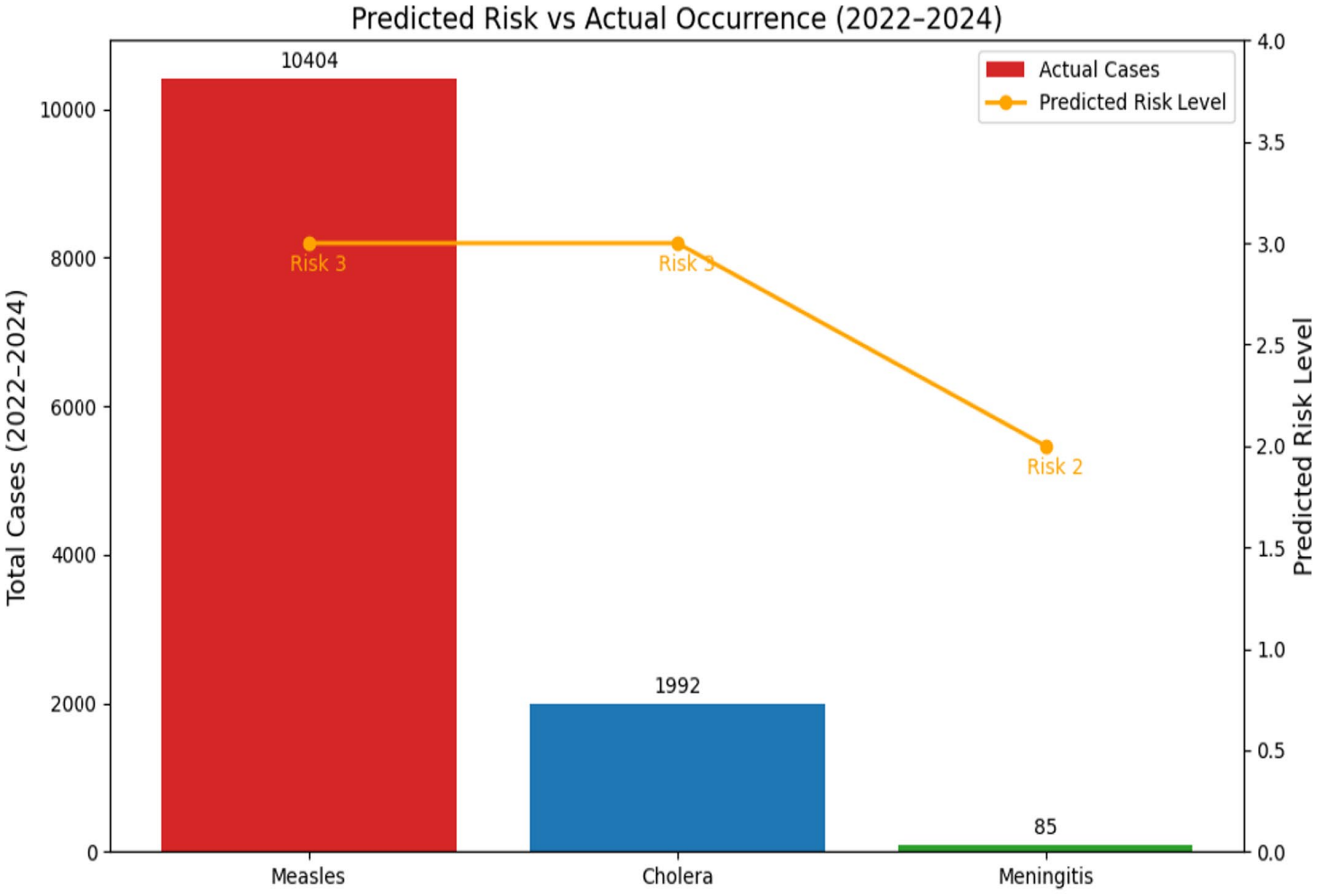
Predicted vs. occurrence in Cameroon (2022–2024). It is noted that though risk level is same for measles and cholera (3), actual case occurrence is different. Meningitis risk level is 2 with and only 85 cases actually occurred.

##### 3.1.2.2 Example of the Central African Republic

See Figures 3, 4.

**Figure 3:**
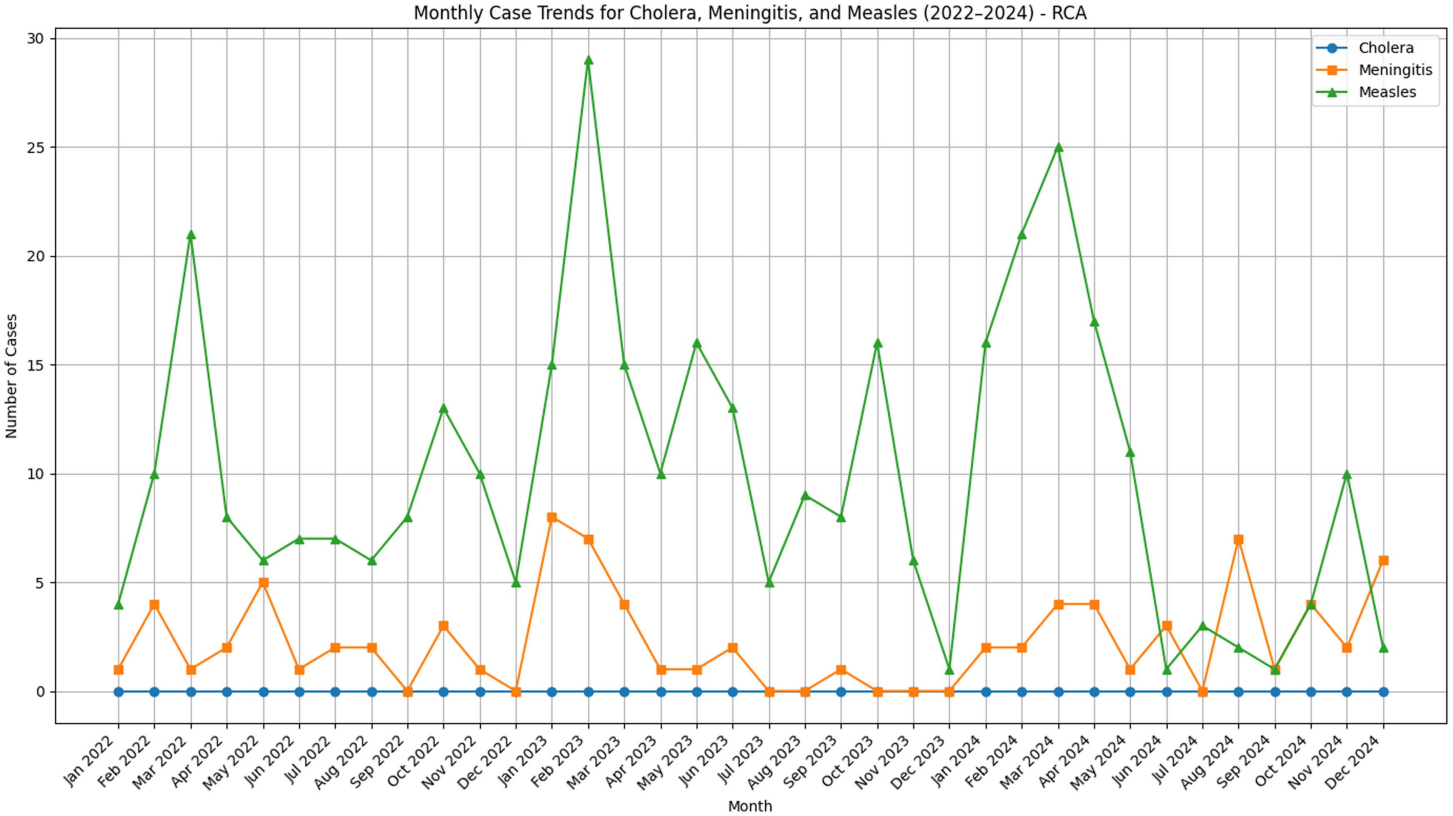
Monthly case trends for cholera, meningitis, and meningitis in the Central African Republic.

**Figure 4:**
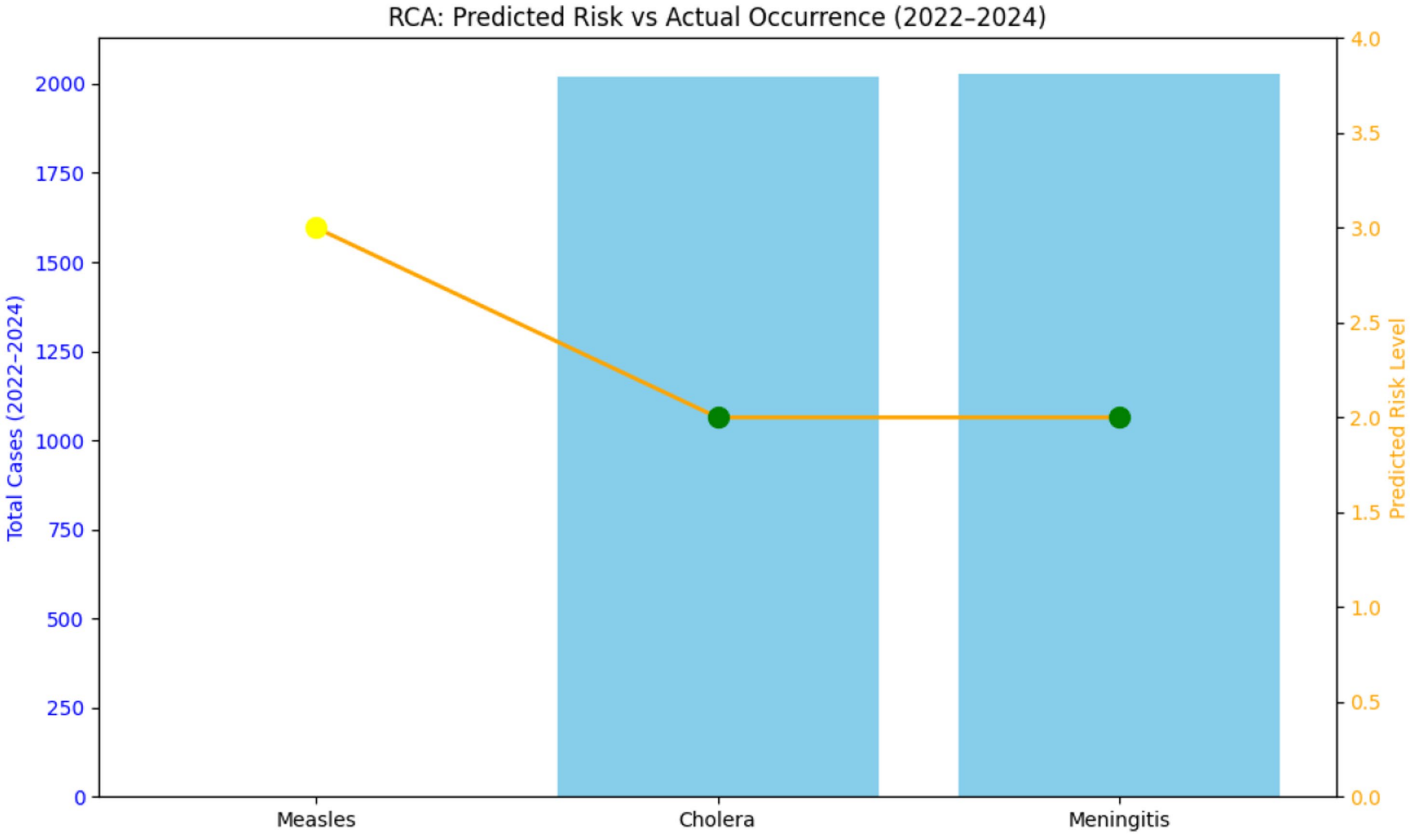
STAR risk classifications and cumulative case counts for measles, cholera, and meningitis in the Central African Republic (2022–2024).

##### 3.1.2.3 Example of Côte d’Ivoire

**Figure 5:**
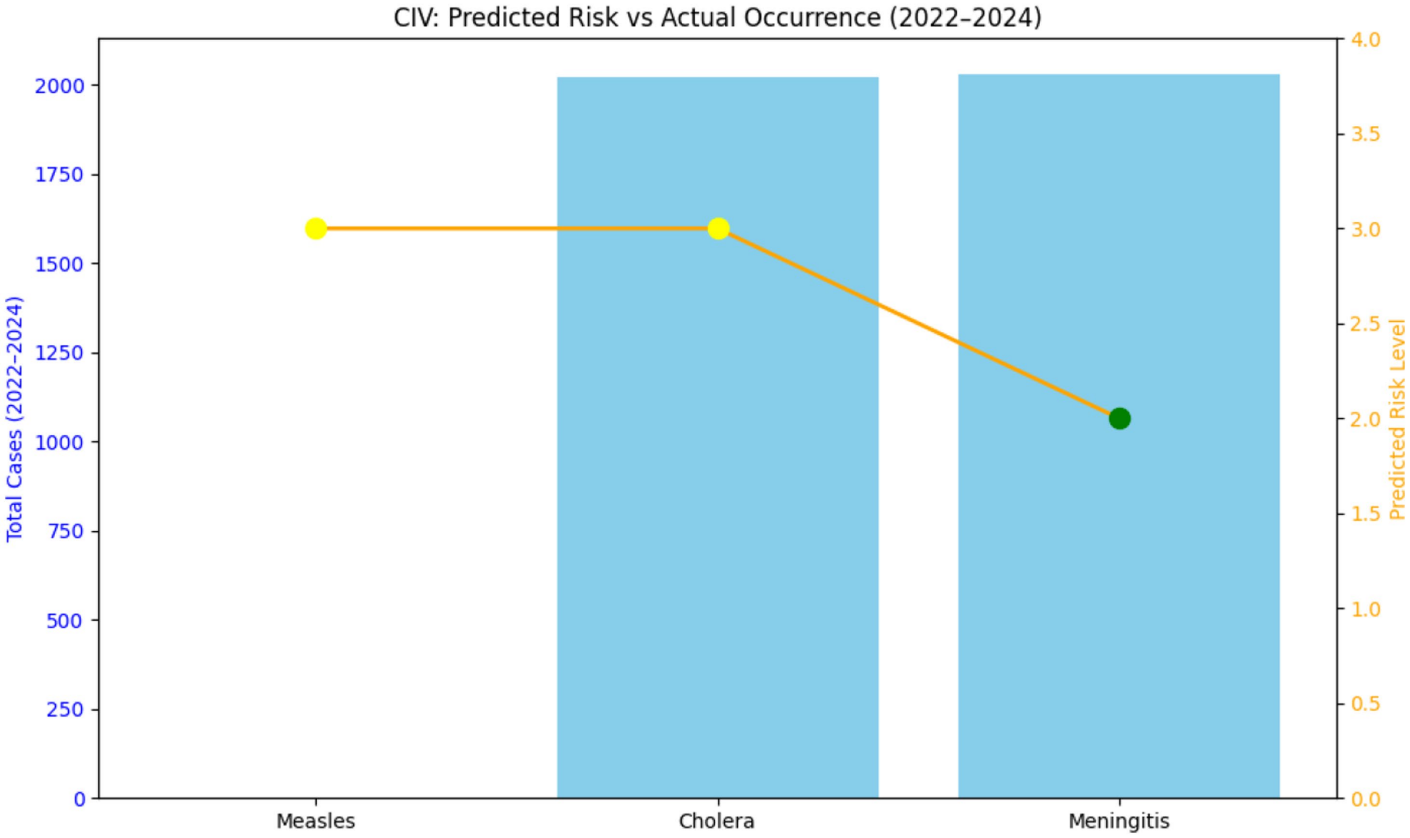
STAR risk classifications and cumulative case counts for measles, cholera, and meningitis in Côte d’Ivoire (2022–2024). This report compares predicted risk levels and actual case occurrences for Cholera, Meningitis, and Measles in Côte d’Ivoire from 2022 to 2024. The analysis features year-by-year breakdowns and trend observations.

**Figure 6:**
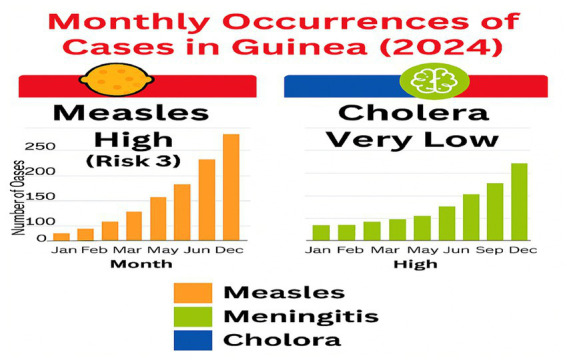
Monthly occurrences of cases in Guinea 2024.

See Figures 5, 6 and Table 5.

**Table 5:** Summary of STAR-predicted risk levels and observed outbreak cases for key hazards in Côte d’Ivoire (2022–2024).

Hazard	Predicted risk level	Actual cases (2022–2024)	Interpretation
Measles	High (3)	2,154	Predicted risk shows general agreement with observed values.
Cholera	High (3)	0	No reported cases despite high risk
Meningitis	Moderate (2)	23	Low but consistent cases

This result shows a descriptive interpretation of agreement.

## 4 Discussion

The findings of this three-year retrospective analysis reveal that the predictive accuracy of the STAR tool varies significantly, correlating primarily with the intrinsic predictability and seasonality of the public health hazards under review, as well as the effectiveness of national coping mechanisms. The discussion section attributes the differences in predictive accuracy for Meningitis compared to Cholera and Measles primarily to the predictability of the disease drivers and the impact of external factors not fully integrated into the STAR tool’s static risk assessment.

### 4.1 Effectiveness for meningitis: high consistency

STAR was highly effective for Meningitis because the disease’s risk factors are well-defined, highly seasonal, and predictable: Strong Seasonality: Meningitis outbreaks in West and Central Africa are strongly tied to the dry season (the “Meningitis Belt”) and predictable climate factors (low humidity, dust, wind). These drivers are relatively stable and can be captured by static models. The disease follows a highly consistent and understood epidemiological pattern across the region, making the Likelihood component of the STAR score inherently more reliable. Moreover, while interventions exist, the seasonal climatic drivers remain dominant and less susceptible to rapid, localized changes, allowing the tool’s assessment to hold true over the monitoring period.

### 4.2 Lack of effectiveness for cholera: low reliability

Cholera predictions were the least reliable because the disease dynamics are highly sensitive to rapidly changing factors, particularly human interventions: There is a high sensitivity to preparedness because cholera risk is quickly and drastically mitigated by effective Water, Sanitation, and Hygiene (WASH) interventions, which are difficult for a static risk assessment tool like STAR to quantify accurately. For instance, the high-risk prediction for Côte d’Ivoire resulted in zero cases, interpreted as a success of proactive, effective preparedness measures that effectively negated the predicted threat. The tool accurately signaled the potential for risk, but the actual outcome was determined by response capacity. Additionally, it is observed that environmental variability with localized factors like rainfall, flooding, population displacement, and rapid urbanization affects contamination and transmission, introducing high variability that the tool struggles to capture in its composite score.

### 4.3 Lack of effectiveness for measles: variable accuracy

Measles predictions showed moderate but variable accuracy because the key driver, immunization coverage, is dynamic and subject to external shocks such as reliance on Dynamic Human Factors: Measles risk is fundamentally driven by immunity gaps in the population. These gaps are caused by constantly changing factors such as routine immunization rates, campaign effectiveness, and service disruption; factors like the COVID-19 pandemic or civil insecurity severely disrupt vaccination campaigns, leading to sudden, unpredictable drops in coverage. The STAR tool’s relatively static inputs often underestimated the risk because it did not fully account for these rapid, catastrophic service delivery collapses that fueled major outbreaks (e.g., the surge in cases in Burkina Faso).

In summary, the high predictability of Meningitis’s environmental drivers made it well-suited for STAR, while the dynamic and intervention-sensitive nature of Cholera and Measles introduced volatility that limited the tool’s raw predictive power. The study demonstrated the highest consistency between STAR’s predicted risk levels (typically Moderate to High in the Meningitis Belt countries) and actual emergency occurrences for meningitis (Figure 4) in Central African Republic. This strong correlation is largely attributed to the disease’s established, non-linear seasonality (peaks in the dry season) and established epidemiological patterns in the African meningitis belt. The results from countries like Burkina Faso (Table 2) affirm that STAR is highly reliable for hazards whose drivers (such as dry season climate) are relatively constant and measurable. This finding aligns with established public health literature, which consistently links meningitis outbreaks in Africa to specific climate and geographical factors (e.g., wind, dust, humidity) that make the hazard’s timing highly predictable for risk modeling (4). Cholera forecasts showed the least reliability, characterized by frequent overprediction and occasional underestimation of outbreak severity. The most significant discrepancy was the overprediction observed in countries like Côte d’Ivoire and Burkina Faso (Table 1), where a high predicted risk resulted in zero or near-zero actual cases. Conversely, the high predicted risk in Cameroon still underestimated the peak severity of the 2022 outbreak, which saw 1992 cases (Table 1) This weak correlation suggests that while STAR accurately captures *vulnerability* factors (e.g., poor sanitation, population density), it struggles to account for the dynamic impact of interventions and real-time environmental changes (5). Cholera dynamics are highly sensitive to Coping Capacity factors like prompt vaccination campaigns and effective Water, Sanitation, and Hygiene (WASH) improvements, which can rapidly decouple the predicted risk from the actual case counts, a challenge confirmed by recent mathematical modeling approaches (6). Measles predictions showed a moderate correlation but high variability, influenced heavily by changing immunization coverage and socio-political factors. Where predictions were accurate (e.g., Cameroon and Côte d’Ivoire), the high-risk classification was validated by major outbreaks with 10,404 and 2,154 cases, respectively (see Figure 2 and Tables 4, 5). Discrepancies arose due to underestimation of outbreaks stemming from low immunization rates and COVID-19-related service disruptions, as seen by the surge in cases in Burkina Faso in 2024. This finding directly reflects the long-term impact of the COVID-19 pandemic, which caused widespread disruption of routine immunization services and contributed to a substantial accumulation of susceptible populations (7). This drop in immunization coverage—a critical component of STAR’s Vulnerability score—was likely not fully captured by the static input data used in the STAR assessments, leading to a consistent underestimation of outbreak size and severity.

Regarding the role of the STAR Tool in Preparedness, it is observed that the cases of overprediction (e.g., zero cholera cases in Burkina Faso despite high risk, Table 3) serve as strong evidence that the Coping Capacity dimension of STAR can effectively mitigate the consequences of high intrinsic risk. The study suggests that STAR is most valuable as a strategic planning tool for hazard prioritization, rather than a precision forecasting instrument. Where STAR identified a high risk (Risk 3), it accurately signaled the need for increased preparedness, which in several cases (cholera control) appears to have successfully prevented the predicted emergency. This demonstrates a successful function of the STAR output: driving preventative action.

To improve accuracy, particularly for dynamic diseases like cholera and measles, the STAR framework requires enhanced integration with real-time, granular data sources related to climate and Environmental Data (to better model evolving conditions), vaccination Coverage Data (to track drops in herd immunity in real time) and cross-border coordination effectiveness (a key factor in the spread of measles and other pathogens).

Between 2022 and 2024, predictive models were assessed for their ability to forecast Cholera, Meningitis, and Measles outbreaks across African countries using the STAR tool, which assigns monthly risk scores based on likelihood, severity, vulnerability, and health system capacity (8, 9).

In Burkina Faso, cholera was predicted to be a moderate risk, but no cases occurred, suggesting either an overestimation or successful prevention. Meningitis predictions aligned with seasonal outbreaks, validating the model, while measles showed a mismatch moderate predicted risk versus a surge in early 2024 indicating the need for recalibration (10).

Regional cholera trends revealed seasonal peaks and disparities, with Guinea and Niger most affected. West Africa recorded over 399,000 cases and 7,000 deaths between 2022 and mid-2024, driven by rains and poor sanitation (11). Ebenezer et al. (6) employed machine learning to categorize countries based on outbreak patterns and estimate indicators such as R₀, thereby supporting the development of tailored interventions. Tang et al. (4) highlighted climate-sensitive factors air pollution, temperature, dust impacting meningitis in the Sahel, reinforcing climate-based predictive models for early warning. Charkhakan and Heravi (12) proposed an integrated risk framework combining probability, impact, and manageability to strengthen preparedness. In Burundi, predicted risks did not match actual cases (zero reported), possibly due to underreporting or surveillance gaps. Nipa and Allen (13) emphasized patch-specific modeling and seasonal calibration to improve prediction accuracy. In Cameroon, cholera predictions underestimated the 2022 outbreak (more than 1,700 cases), followed by a sharp decline. Meningitis remained low but persistent, while measles predictions were accurate, especially during the 2023 peak (14). Ebenezer et al. (6) applied a Bayesian framework and unsupervised learning to classify outbreak dynamics, thereby reinforcing the need for localized risk assessment in STAR. Bhavithra and Devi (7) developed a time-varying SEIR model for measles, supporting STAR’s objectives of risk identification, contextual analysis, and response planning. In CAR, cholera was predicted as a moderate risk but absent, likely due to WASH interventions. Meningitis aligned with seasonal dry periods, and measles predictions were validated by a 2023 peak (15). In Côte d’Ivoire, measles surged in 2024, confirming high-risk predictions, while cholera (high-risk forecast) did not occur. Meningitis remained moderate with seasonal peaks, aligning with STAR’s classifications. In the Republic of the Congo, WHO reported a suspected triple outbreak of typhoid, shigellosis, and cholera in 2023. Despite cholera being predicted as a very high risk, only 21 confirmed cases occurred, indicating a localized outbreak and gaps in surveillance. The response included multisectoral coordination and community engagement, reflecting STAR principles (16, 17). Overall, STAR proved useful for hazard prioritization but exhibited limitations, including static inputs and underestimation of outbreaks. Recommendations include recalibrating scoring, integrating real-time surveillance, enhancing seasonality modeling, and improving transparency (15–22). In Gabon, the STAR framework was applied to prioritize hazards. WHO’s Midterm Results Report (2024–2025) confirmed measles as a very high-risk hazard, prompting intensified immunization campaigns, stockpiling of emergency supplies, and enhanced surveillance. Cholera was assessed as very low risk, with preparedness focused on hygiene promotion and rapid response readiness, supported by WHO logistics hubs in Dakar and Nairobi. Meningitis was classified as high risk due to its seasonal nature and potential for cross-border transmission, necessitating cross-border coordination, vaccination campaigns, and contingency planning. These actions align with STAR’s emphasis on vulnerability, burden, and preparedness (18). In Guinea, 2024 outcomes validated STAR predictions. Measles experienced a major outbreak, confirming its high-risk classification and linking the resurgence to declining immunization coverage and post-pandemic disruptions. Cholera remained absent, consistent with its very low-risk prediction, suggesting effective preventive measures or unfavorable environmental conditions. Meningitis showed moderate activity, partially aligning with its high-risk classification, reinforcing the need for seasonal vaccination and cross-border coordination. These outcomes demonstrate STAR’s utility in hazard prioritization and resource allocation (19). In Mali, 2022 outcomes aligned closely with STAR predictions. Measles outbreaks confirmed its high-risk classification, linked to low immunization coverage, with only 70% of children receiving the first dose of the measles vaccine. Cholera did not occur, as predicted, matching its low-risk forecast, likely due to effective preventive measures and possibly favorable environmental conditions. Meningitis showed consistent presence with seasonal peaks, supporting its classification as a moderate to high-risk hazard and justifying continued vaccination campaigns and surveillance efforts (15). In Nigeria, statistical analysis revealed a strong correlation between predicted and actual cholera cases in 2024, indicating high model accuracy. Measles exhibited a moderate correlation but high variability, suggesting unpredictability in outbreaks, whereas meningitis lacked sufficient data for meaningful analysis. Recommendations included improving data quality, adopting advanced predictive models, and integrating external data sources such as climate and mobility patterns (20). Across all countries, several cross-cutting themes emerged. Meningitis predictions were generally accurate due to strong seasonality, whereas measles posed challenges due to variability and underprediction. Cholera predictions often overestimated risk or failed to capture localized outbreaks. The report emphasized the need for dynamic modeling, real-time data integration, and feedback loops to refine forecasting accuracy (21). The STAR tool itself was evaluated in detail. Its strengths include a structured risk assessment framework, support for prioritization, and multi-hazard capability. However, limitations were noted, such as underestimating outbreaks, reliance on static inputs, and limited geographic granularity. Recommendations for improving STAR included recalibrating scoring parameters, integrating real-time surveillance, enhancing seasonality modeling, and increasing transparency (22).

Overall, aligning predictive models with outbreak data is critical for preparedness. The report recommends implementing improved data systems, training in forecasting tools, and cross-sector collaboration to enhance early warning systems (15–22).

## 5 Limitations

While this study provides valuable insights into the alignment between STAR-predicted risk levels and observed outbreak patterns, a significant limitation is the absence of a formal statistical criterion for classifying actual case counts into risk categories or quantifying agreement. Interpretations were based on descriptive comparisons rather than inferential analysis, which limits objectivity and reproducibility. Future research should establish verifiable thresholds for mapping case numbers to STAR risk levels and apply statistical measures, such as correlation coefficients, Kappa statistics, or paired-comparison tests, to strengthen methodological rigor. Incorporating these approaches will ensure that conclusions are supported by quantitative evidence and enhance STAR’s predictive utility as a strategic planning tool.

## 6 Conclusion and recommendations

This study aimed to assess the STAR’s predictive accuracy and practical relevance (Strategic Tool for Assessing Risks) framework by comparing its health hazard classifications with actual emergency events across West and Central Africa from 2022 to 2024. The results provide a detailed understanding of STAR’s performance and potential areas for improvement.

1 Comparison of STAR risk classifications with actual outbreak data

The analysis revealed that STAR’s predictions were most accurate for seasonal and geographically consistent hazards like meningitis, where actual outbreaks closely mirrored forecasted risk levels. However, the tool showed mixed results for measles and cholera either underestimating or overpredicting outbreaks highlighting limitations in accounting for dynamic factors such as vaccination coverage, environmental changes, and population mobility.

2 Evaluation of preparedness measures in mitigating predicted risks

In countries like Burkina Faso and Gabon, strong preparedness measures (e.g., vaccine pre-positioning, hygiene promotion, and cross-border coordination) effectively mitigated predicted risks, resulting in fewer or no cases despite high-risk forecasts. This highlights the importance of combining risk assessments with solid preparedness actions to prevent or contain outbreaks.

3 Identification of Patterns and Gaps in STAR’s Predictive Accuracy

The study revealed consistent patterns in STAR’s performance.

- Strengths: reliable forecasting of meningitis due to its seasonal nature and established epidemiological patterns.
- Gaps: inadequate sensitivity to emerging or localized outbreaks, especially for cholera and measles, where socio-political and environmental factors played a significant role.
- Limitations: static inputs, lack of real-time data integration, and limited geographic granularity reduced the tool’s responsiveness and precision.

While STAR is not a statistical forecasting tool, it remains a strategic planning instrument that supports national health emergency preparedness. Its participatory, multi-sectoral approach fosters country ownership and facilitates resource prioritization. The study recommends integrating real-time surveillance data, refining scoring parameters, and improving geographic resolution to enhance its utility. STAR can significantly strengthen the region’s early warning systems and emergency response planning when complemented with dynamic data and cross-sectoral collaboration.

These findings highlight the need to improve the STAR tool continually. It should include real-time surveillance data, increase geographic detail, and build strong feedback systems to boost its predictive accuracy. STAR should be viewed not just as a statistical forecast but as a strategic planning resource that can significantly strengthen national and regional health emergency preparedness when combined with dynamic data and cross-sector collaboration.

To improve STAR’s predictive accuracy and utility, it would be useful to recalibrate scoring parameters using recent outbreak data, integrate real-time surveillance and external data (e.g., climate, mobility), enhance seasonality modeling for diseases with known temporal patterns, increase geographic granularity to district or health zone level, establish feedback loops for continuous model refinement, and improve transparency in how scores are calculated and interpreted.

4 Potential biases and study limitations

- The study notes that the accuracy of the correlation between predicted risk and actual cases is highly dependent on the quality and completeness of national surveillance data, leading to the risk of underreporting (The most direct evidence of this is seen in cases where reported cases did not match a predicted risk. For instance, in Burundi, predicted risks did not match the zero reported cases, which the authors acknowledge may have been “possibly due to underreporting or surveillance gaps”) and gaps in Surveillance (Similarly, in the Republic of Congo, a “Very High” risk prediction for cholera resulted in only 21 confirmed cases, which was interpreted as indicating a small, localized outbreak and potential “gaps in surveillance”).

Concerning biases from Variations in National Workshops (Static Inputs), the STAR tool’s reliance on inputs gathered during periodic national workshops or assessments creates a limitation related to static data and variations in the assessment process such as reliance on Static Inputs: The core limitation of the STAR tool noted in the discussion is its “reliance on static inputs” and the “inability to capture real-time, dynamic factors.” The risk scores are based on the situation *at the time of the workshop* and may not capture rapid, intervening changes and failure to Capture Dynamic Factors: This issue led to discrepancies, particularly for measles and cholera. To mitigate the organizational bias, the study implemented a robust procedural safeguard. For instance, to ensure independent verification, the Actual Emergency Occurrences (case data) were verified using standardized, third-party data sources such as WHO reports under the International Health Regulations (23) and externally audited humanitarian reports. Furthermore, the entire correlation analysis was subjected to an external review process by an independent epidemiologist (unaffiliated with WHO or the study countries) who “confirmed the calculated correlation coefficients and conclusions” prior to the manuscript’s submission.

This dual-layered approach, using external data for validation and an external reviewer for the analysis, ensures that the core findings regarding the correlation between STAR predictions and real-world events are independently verified, thereby mitigating the risk of organizational bias.

## Data Availability

The datasets presented in this study can be found in online repositories. The names of the repository/repositories and accession number(s) can be found in the article/supplementary material.
